# Landmine Press Kinematics Measured with an Enhanced YOLOv8 Model and Mathematical Modeling

**DOI:** 10.3390/s26041161

**Published:** 2026-02-11

**Authors:** Rui Zhao, Rong Cong, Ruijie Zhou, Kelong Lin, Jianke Yang, Tongchun Kui, Jiajin Zhang, Ran Wang, Rou Dong

**Affiliations:** 1 College of Physical Education, Yunnan Agricultural University, Kunming 650201, China; 2023055@ynau.edu.cn (R.Z.); 2021022@ynau.edu.cn (R.C.); regina_chou@ynau.edu.cn (R.Z.); 2024240819@stu.ynau.edu.cn (K.L.); yangjianke@ynau.edu.cn (J.Y.); 2023315330@stu.ynau.edu.cn (T.K.); 2 Center for Sports Intelligence Innovation and Application, Yunnan Agricultural University, Kunming 650201, China; zjjclc@ynau.edu.cn; 3 School of Athletic Performance, Shanghai University of Sport, Shanghai 200438, China

**Keywords:** landmine press, kinematics, YOLOv8, mathematical modeling

## Abstract

**Highlights:**

**What are the main findings?**

- An enhanced YOLOv8-OBB vision system provides a markerless approach for tracking landmine press kinematics, demonstrating strong agreement with a commercial linear transducer across velocity and power metrics.
- The system shows consistent measurement stability under high-load conditions, where sensor-based devices are susceptible to electromechanical noise and drift, despite exhibiting a predictable overestimation in velocity.

**What are the implications of the main findings?**

- This computer vision approach offers a practical and non-invasive alternative to attached sensors for monitoring strength training, suitable for both field and laboratory environments.
- The study illustrates how advanced object detection models can be adapted to develop specialized “vision sensors” for biomechanical applications, bridging computer vision and sports science.

**Abstract:**

The landmine press is a reliable and valid test for assessing upper-body push strength. However, its application is constrained by the limitations of current mainstream monitoring technologies, such as linear position transducers (LPTs). These devices require physical attachment to the barbell, they rely on proprietary software, and their measurement accuracy can degrade under high-load conditions due to sensor drift and electromechanical noise. To address these limitations, this study developed a markerless, non-contact, and vision-based system using an enhanced YOLOv8-OBB model and a mathematical modeling framework to measure four kinematic indicators during the concentric phase of the landmine press. By integrating a polarized self-attention mechanism, an improved C3k2 module, and an optimized SPPF structure, the system significantly enhanced detection accuracy and robustness for the small targets at both ends of the barbell, achieving an mAP@0.5 of 0.995 on the test set. A method comparison study was conducted against a widely used LPT device (GymAware) across four loads (20–35 kg) in 247 trials. The results showed strong correlations (r > 0.85) for peak velocity, mean velocity, peak power, and mean power. Although the vision-based method systematically overestimated velocity metrics, the bias was predictable. Notably, under the highest load (35 kg), where LPT limitations are pronounced, the vision system demonstrated comparative stability, suggesting its potential advantage in mitigating sensor-related errors. The findings demonstrate that this vision-based system offers a reliable and practical alternative for monitoring landmine press kinematics, suitable for both training and scientific research.

## 1. Introduction

The landmine press is a widely used, reliable and valid training exercise for athletes to develop unilateral strength and upper-body power [1,2]. This exercise has direct transferability to athletic movements such as punching in sports like boxing and MMA [2,3]. This exercise is performed using a specialized apparatus, a landmine, where one end of a barbell is anchored to the floor, allowing the other end to move freely in an arc [2,4]. Effective monitoring of its kinematics, specifically velocity, force and power metrics during the concentric phase, is essential for performance assessment and 1RM prediction [5].

Currently, the primary method for monitoring barbell kinematics in training environments is the Linear Position Transducer (LPT). Representative devices such as the GymAware RS (GYM; Kinetic Performance Technologies, Canberra, Australia) employ a cable attached to the moving end of the barbell, from which velocity, force and power are derived based on the vertical displacement of the cable [5,6]. LPTs exhibit recognized limitations: (1) their accuracy can degrade under high load due to sensor drift and noise accumulation [5,6]; (2) their setup requires physical attachment and manual calibration [6]; (3) they are inherently less suited for accurately tracking non-vertical, rotational movements like the landmine press [5].

Concurrently, computer vision (CV) has emerged as a promising, non-contact alternative for sports kinematics and biomechanics. Recent studies have validated the feasibility of video-based systems for measuring barbell kinematics in controlled settings. For example, smartphone applications have shown strong agreement with 3D motion capture in tracking vertical barbell displacement and velocity during exercises such as squat and snatch [7,8]. Automated image-processing pipelines using homography transformation have further enabled real-time and unattended barbell tracking in fixed-machine equipment [9]. Additionally, deep learning models such as Keypoint RCNN and YOLOv7 have been successfully applied to concurrently estimate athlete poses and barbell trajectory in Olympic weightlifting [10,11]. However, existing CV approaches have largely focused on linear or vertical barbell paths. The landmine press, with its pronounced rotational arc and angular displacement, presents a distinct challenge, requiring precise detection of both barbell ends and accurate orientation estimation, tasks for which conventional horizontal-bounding box detection is insufficient.

To bridge this gap, this study proposes a novel vision-based system specifically designed for the landmine press. Our approach integrates an enhanced YOLOv8-OBB (oriented bounding box) model for precise detection of the barbell ends with mathematical modeling to calculate key kinematic indicators. Recognizing the practical need for a manageable model, our calculations are based on a simplified planar rigid-body assumption of the barbell rotation, providing a practical estimate of the kinematics. The purposes of this study are as follows: (1) to develop a CV based algorithm for measuring kinematic indicators during the concentric phase of the landmine press, using an enhanced YOLOv8-OBB model and mathematical modeling; (2) to conduct a method comparison study against a widely used LPT device (GymAware) to evaluate its agreement and performance across a range of loads.

## 2. Materials and Methods

### 2.1. Data Collection and Annotation

This study constructed a self-collected dataset for model training and validation. Two healthy male volunteers (training experience > 3 years) performed the standing landmine press under controlled gym conditions. A camera (Sony FDR-AX60 (Sony Corporation, Tokyo, Japan), 50 fps, 1080p) was fixed at a 90° frontal view to record the movements. In total, 15 videos were captured across four loads (20, 25, 30, 35 kg), ensuring that both the moving and fixed ends of the barbell remained within the frame throughout each trial.

#### 2.1.1. Dataset Composition and Partitioning

The final annotated dataset comprised 1120 frames, which were divided into training, validation, and test sets with a ratio of 7:2:1, respectively. The distribution of frames across participants and loads was approximately balanced, with each participant contributing a similar number of frames under each load condition. To prevent data leakage and ensure model generalizability, the dataset was partitioned at the video level such that frames from the same participant and load condition were exclusively assigned to one of the three sets. No subject overlap existed between the training and test sets.

#### 2.1.2. Annotation Protocol

A single researcher labeled the moving and fixed ends of the barbell with oriented bounding boxes (OBBs) using the X-AnyLabeling (version 2.4.2) tool. To ensure annotation consistency, a random sample of 100 frames was independently cross-checked by a second researcher. The root mean square error (RMSE) of the annotation coordinates was less than 2.5 pixels, confirming high inter-rater reliability.

#### 2.1.3. Dataset Preprocessing and Filtering

To ensure the reliability of the kinematic indicators (velocity, acceleration) extracted from the raw video coordinate data and to suppress the amplification effect of noise during numerical differentiation, this study adopted a tandem data filtering pipeline, with all signal processing implemented in Python 3.12.7 (Using the Scipy library 1.13.0). The raw 2D coordinate data were first smoothed using a Savitzky–Golay filter (window size = 9, polynomial order = 3) to preserve high-frequency features while minimizing phase distortion. For velocity calculation, input signals (angle θ(t) and vertical position y(t)) were pre-smoothed with the same filter, and the resulting velocity v(t) was further processed with a zero-phase Butterworth low-pass filter (cutoff frequency = 6 Hz) to eliminate phase delay and ensure accurate peak timing. Acceleration was derived by differentiating the smoothed velocity signal and then filtering it with the same Butterworth low-pass filter to reduce measurement noise.

### 2.2. An Enhanced YOLOv8-OBB Object Detection Approach

In this study, we selected YOLOv8 as the baseline object detection framework due to its demonstrated balance between inference speed and accuracy. Compared to earlier versions such as YOLOv5, YOLOv8 offers improved feature fusion mechanisms and a more efficient architecture for small object detection [12]. While subsequent versions (e.g., YOLOv9, YOLOv10) have further advanced accuracy, they often do so at the cost of increased computational complexity and slower inference speed [13]. Therefore, YOLOv8 represents a well-balanced choice that maintains high precision for small targets, such as the barbell ends in a cluttered gym environment.

Based on the YOLOv8-OBB framework, this study employs an enhanced YOLOv8-OBB architecture to optimize the model for the precise detection of two small targets: the moving and fixed ends of the landmine.

**Figure 1:**
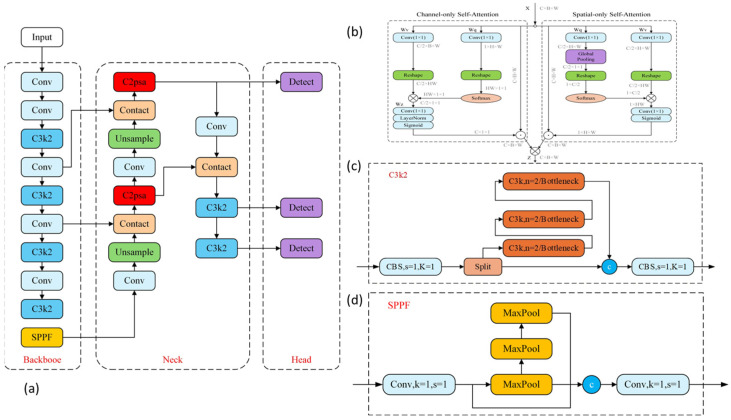
Enhanced YOLOv8-OBB architecture diagram. (**a**) Backbone–Neck–Head framework of the enhanced YOLOv8-OBB, (**b**) detailed structure of the PSA module, the symbol ⊗ denotes matrix multiplication for similarity computation, while ⊙ denotes element-wise multiplication (Hadamard product) for applying attention weights to features, (**c**) detailed structure of the C3k2 module, (**d**) detailed structure of the SPPF module.

The model was enhanced with three key improvements for small-object detection. First, the polarized self-attention (PSA) module. During the press, the athlete’s torso and limbs produce accompanying movements, creating a complex dynamic background. The “filter and focus” mechanism of the PSA module can eliminate these distractions and concentrate computational resources on the small yet critical regions of interest at both ends of the barbell [14]. The structure of the PSA module is illustrated in Figure 1b, which highlights its dual-path design for channel and spatial attention, enabling selective feature enhancement.

Second, the improved C3k2 module. The barbell undergoes a wide range of angular variation during the press. To address the potential loss of angular signal that may occur when using a single-feature-path approach to capture such a broad range of rotational changes, the standard C3 module was replaced with the C3k2 module. The heterogeneous fusion paths of C3k2 enable the parallel processing of features at different levels of abstraction: local fine-grained features are leveraged to detect subtle initial angular displacements, while global contextual features help stabilize angle estimation under challenging conditions such as complex backgrounds or motion blur [15]. As shown in Figure 1c, the C3k2 module incorporates two parallel convolutional branches with different kernel sizes (k = 1 and k = 3) to capture multi-scale features.

Third, the improved SPPF (Spatial Pyramid Pooling Fast) module. Accurate determination of the moving end’s instantaneous state (e.g., concentric phase, peak instant) requires the model to understand the brief motion context surrounding a frame. the SPP module was substituted with a faster SPPF module, which uses serial 5 × 5 max-pooling layers to capture contextual information across varying receptive fields for each target with high computational efficiency [14]. Figure 1d illustrates the serial pooling design of SPPF, which reduces latency while maintaining multi-scale feature integration capability [16].

The overall architecture of the proposed Enhanced YOLOv8-OBB model is depicted in Figure 1a, with key sub-modules detailed in panels (b)–(d).

The enhanced YOLOv8-OBB model was trained on the LP dataset for 120 epochs, initialized with pre-trained weights. Training used a 960 × 960 input size, a batch size of 4 (effective size 64 via gradient accumulation), and an automated optimizer (learning rate set to 0.01). Data augmentation included HSV, mosaic (disabled last 10 epochs), and Randaugment. Key metrics were precision, recall, mAP@0.5, and mAP@0.5:0.95. All experiments were run on a single GPU.

### 2.3. Mathematical Modeling

#### 2.3.1. Model Assumptions

The kinematic model developed in this study is based on several simplifying assumptions to enable markerless motion analysis from 2D video. The barbell is treated as a rigid body undergoing a primarily planar rotation in the sagittal plane, with the fixed end of the landmine considered a stationary pivot point. This assumption neglects any compliance or micro-motion of the anchor system, as well as grip-related micro-movements and out-of-plane motion components, which are assumed negligible for the purpose of velocity and power estimation. For force and power calculations, the barbell mass is considered concentrated at its ends, neglecting mass distribution along the bar and friction effects. These assumptions are common in kinematic modeling of barbell exercises and allow the proposed vision-based system to remain generalizable across different training environments, equipment setups, and athlete populations without 3D motion capture systems or force plate instrumentations [8,11].

#### 2.3.2. Identifying Moving and Fixed Ends and Establishing Coordinates

This study used deep learning for rotated-rectangle detection to identify landmine bars’ postures. For each detected bounding rectangle, its four corners were arranged into a coordinate matrix:

(1) $$Points=\left[\begin{array}{ll} x_{1} & y_{1}\\ x_{2} & y_{2}\\ x_{3} & y_{3}\\ x_{4} & y_{4} \end{array}\right]$$

The principal axis orientation of the rotated rectangle was determined by calculating the Euclidean distance between adjacent corner points.

(2) $$L_{1}={∥\begin{array}{c} P_{1}-P_{2} \end{array}∥}_{2}$$

(3) $$L_{2}={∥\begin{array}{c} P_{2}-P_{3} \end{array}∥}_{2}$$

$L_{1}$ and $L_{2}$ denote the lengths of two adjacent sides. The longer side was defined as the landmine’s principal axis. The axis endpoints were calculated as the midpoints of the two opposing longer sides:

(4) $$\left\{\begin{array}{ll} M_{1}=\frac{P_{1}+P_{4}}{2} & \mathrm{if}\;L_{1}>L_{2}\\ M_{2}=\frac{P_{2}+P_{3}}{2} & \mathrm{if}\;L_{1}>L_{2}\\ M_{1}=\frac{P_{1}+P_{2}}{2} & \mathrm{otherwise}\;\\ M_{2}=\frac{P_{3}+P_{4}}{2} & \mathrm{otherwise}\; \end{array}\right.$$

Based on the exercise’s biomechanics, this study distinguishes dynamic and static ends by their vertical (Y-axis) position. In the image (origin top-left), the fixed end is typically lower, while the moving end is higher. The discriminant function is as follows:

(5) $$\mathrm{MovingEnd}=\left\{\begin{array}{ll} M_{1} & \mathrm{if}\;M_{1}^{y}<M_{2}^{y}\\ M_{2} & \mathrm{otherwise}\; \end{array}\right.$$

(6) $$\mathrm{FixedEnd}=\left\{\begin{array}{ll} M_{2} & \mathrm{if}\;M_{1}^{y}<M_{2}^{y}\\ M_{1} & \mathrm{otherwise} \end{array}\right.$$

$M_{i}^{y}$ represents the Y-coordinate. This criterion utilizes the physical constraint that the fixed end’s spatial position is relatively stable during the exercise.

A scale calibration factor was introduced to convert pixels to physical coordinates. First, the average bar length was calculated as follows:

(7) $$L_{\mathrm{pixel}}=\frac{1}{N}\sum_{i=0}^{N-1}{∥\begin{array}{c} {\mathrm{MovingEnd}}_{i}-{\mathrm{FixedEnd}}_{i} \end{array}∥}_{2}$$

Given the actual bar length, the scale factor s was calculated as

(8) $$s=\frac{L_{\mathrm{real}}}{L_{\mathrm{pixel}}}$$

The final trajectory in the physical coordinate system was

(9) $${\mathrm{trajectory}}_{\mathrm{physical}}=s\times {\mathrm{trajectory}}_{\mathrm{pixel}}$$

It should be noted that the vision-based system uses the full barbell length ($L_{\mathrm{real}}$ = 2.20 m) for angular velocity conversion, whereas the GymAware system measures from a point 15 cm from the moving end, resulting in an effective radius of approximately 2.05 m [15]. This difference in measurement radius contributes to a systematic bias in linear velocity estimates between the two systems.

#### 2.3.3. Mathematical Calculation of Kinematic Indicators

**Figure 2:**
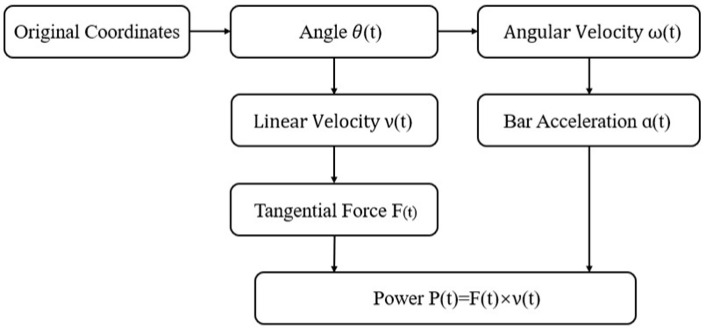
Mathematical calculation methodology for kinematic indicators. The pipeline begins with the barbell angle $\theta \left(t\right)$, calculates angular velocity $\omega \left(t\right)$ and linear velocity $v\left(t\right)$, and then derives force $F\left(t\right)$ and power $P\left(t\right)$ based on inertial and gravitational components. All signals are filtered for reliability, yielding the peak and mean velocity and power outputs for the concentric phase under a planar rigid-body assumption.

In accordance with the methodology depicted in Figure 2, the estimated kinematic indicators of the moving end relative to the stationary end of the barbell were calculated through mathematical modeling.

First, the real-time angle *θ* between the barbell and the horizontal plane was calculated. Given the fixed-end coordinates $\left(X_{f},Y_{f}\right)$ and the moving-end coordinates $\left(X_{m\left(t\right)},Y_{m\left(t\right)}\right)$, the angle was computed using the four-quadrant inverse tangent function.

(10) $$\theta \left(t\right)=arctan2\left(y_{m}\left(t\right)-y_{f},x_{m}\left(t\right)-x_{f}\right)$$

To eliminate angular jumps, a phase unwrapping algorithm was applied:

(11) $$\theta_{\mathrm{unwrapped}}\left(t\right)=\theta \left(t\right)+2\pi \cdot k\left(t\right)$$

where $k\left(t\right)\in Z$ satisfies $\left|\theta_{\mathrm{unwrapped}}\left(t\right)-\theta_{\mathrm{unwrapped}}\left(t-1\right)\right|\leq \pi$.

The angular velocity ω(t) was computed using the central difference method, following smoothing with a Savitzky–Golay filter:

(12) $$\omega \left(t\right)=\frac{d\tilde{\theta }}{dt}\approx \frac{\tilde{\theta }\left(t+\Delta t\right)-\tilde{\theta }\left(t-\Delta t\right)}{2\Delta t}$$

The angular acceleration α(t) was obtained by differentiating the angular velocity with respect to time:

(13) $$\alpha \left(t\right)=\frac{d\omega }{dt}\approx \frac{\omega \left(t+\Delta t\right)-\omega \left(t-\Delta t\right)}{2\Delta t}$$

where Δt = 1/fps is the sampling interval.

The tangential linear velocity v(t) was determined based on the product of the angular acceleration α(t) and the barbell length L:

(14) $$v\left(t\right)=L\cdot \omega \left(t\right)$$

The barbell length L, obtained through calibration, was 2.2 m in this study for the Olympic barbell.

The estimated tangential force F(t) comprises both inertial and gravitational components:

(15) $$F\left(t\right)=mL\alpha \left(t\right)+mg\sin\theta \left(t\right)$$

where m denotes the total barbell load, defined as the sum of the barbell’s mass and the attached weight plates.

The calculated mechanical power was computed as follows:

(16) $$P\left(t\right)=F\left(t\right)\cdot v\left(t\right)$$

The terms “estimated” and “calculated” are used to emphasize that force and power outputs are derived from the model described above, rather than directly measured by force plates or other direct sensing instruments.

#### 2.3.4. Division of the Concentric Phase

To assess concurrent validity with the GymAware device, the concentric phase recognition algorithm in this study was aligned with the method described in GymAware [15]. It is defined as the movement interval during which the barbell overcomes gravity and moves upward with an acceleration. The identification of the concentric phase must adhere to the following two criteria.

1. Direction Criterion: The velocity signal must be positive (v(t)>0), ensuring an upward movement direction.
2. Significance Criterion: The velocity magnitude must exceed a 0.5 m/s threshold to filter noise and ensure genuine mechanical output. Points meeting this and the angle criterion were marked as candidates. The algorithm then merged consecutive candidates into intervals. To prevent misclassifying transient fluctuations as valid movements, each interval had to last at least 5 frames (~0.1 s at 50 fps); shorter intervals were discarded as noise.

#### 2.3.5. Values During the Concentric Phase

The peak value within the concentric phase was detected using a maximum filter followed by a smoothing procedure.

(17) $$X_{peak}=max\left(maximum\_\right.filter1d\left.\left(SG(X(t)),k=5\right)\right)$$

Specifically, the algorithm applied a one-dimensional maximum filter with a window size of 5, and then smoothed the output using a Savitzky–Golay filter (denoted as SG()).

The mean value for the concentric phase was calculated using a time-weighted average, as defined by the following formula:

(18) $$\bar{X}=\frac{1}{T_{c}}\int_{t_{s}}^{t_{e}}X\left(t\right)dt\;\;T_{c}=\left(t_{e}-t_{s}\right)\cdot \Delta t$$

### 2.4. Method Comparison Experiment

This section describes a method comparison study between the proposed vision-based system and a commercially available linear-position transducer (GymAware). The study is designed to evaluate the agreement between the two measurement systems under various loading conditions, rather than to validate either system against a gold standard. GymAware was selected as the comparator due to its widespread adoption in strength training monitoring and its established use in prior landmine press research [5,17].

#### 2.4.1. Sample Size

A priori sample size calculation was performed using G*Power (version 3.1.9.7). The test family “*t* tests” with the statistical test “Means: Difference between two dependent means (matched pairs)” was selected. Cohen’s d was set to 0.4, based on the effects observed in previous studies by Balsalobre-Fernández et al. [8], involving barbell displacement measurement during a back squat with optical cameras, and Achermann et al. [18], involving barbell velocity measurement during the snatch using a vision-based method. The significance level (α) was set at 0.05, and the statistical power (1-β) was set at 0.80. The a priori analysis indicated that a sample of 52 paired measurements was required to detect a significant difference.

#### 2.4.2. Participants

24 male collegiate athletes from the School of Physical Education at Yunnan Agricultural University were recruited as participants (age = 21.3 ± 2.2 years, training experience = 3.2 ± 0.8 years). All participants met the following inclusion criteria: (1) no upper- or lower-body injuries within the past six months; (2) more than two years of strength and conditioning training experience, with a training frequency of more than three sessions per week over the recent three months; (3) familiarity with the landmine press movement; and (4) right-hand dominance.

#### 2.4.3. Experimental Design

All trials were conducted under controlled gym conditions with fixed camera placement and consistent lighting to ensure measurement consistency and minimize environmental variability. Upon arrival at the gym, participants warmed up for 15 min standardly. After a 3 min rest, participants performed the landmine press test in a split stance (left leg forward, right leg back, with toes pointing forward) with incremental loads of 20 kg, 25 kg, 30 kg, and 35 kg. At each load, 3 reps were completed, resulting in a maximum of 12 trials per participant. Testing was terminated if a participant failed to complete a repetition at a given load. During the press, participants were required to fully grip the moving end. Participants were instructed to press the barbell from the moving end close to the deltoid muscle.

A Sony FDR-AX60 (Sony Corporation, Tokyo, Japan) camera recorded the entire landmine press test at 1080p and 50 Hz. The camera was fixed in a position providing a direct 90° frontal view. Care was taken to ensure that the moving and fixed ends of the barbell are included in every frame.

A GymAware RS (GYM; Kinetic Performance Technologies, Canberra, Australia) collected barbell kinematic data synchronously. The device was set up according to the instruction manual [11]: fix the Velcro at 15 cm from the end of the moving end, fix the sensor to the ground using weight plates, and ensure the cable is perpendicular to the ground. The “Landmine Press (R)” mode was selected for recording. The device was zeroed before each trial. The tests and video recording were both conducted in the indoor gym of Yunnan Agricultural University.

The final dataset comprised 247 valid trials distributed across loads as follows: 20 kg (*n* = 72), 25 kg (*n* = 63), 30 kg (*n* = 56), and 35 kg (*n* = 56). These trials were used exclusively for method comparison and were not included in the training or validation of the object detection model described in Section 2.2, ensuring no data leakage between model development and system evaluation.

### 2.5. Statistical Analysis

The following analyses aim to evaluate both the correlation and the agreement between the vision-based method and the GymAware system. It is important to distinguish between these two concepts: a high correlation indicates a strong linear relationship between the measurements from the two systems, whereas good agreement suggests that the two methods can be used interchangeably in practice [19,20,21]. Therefore, we employed a combination of correlation analysis, regression methods accounting for measurement error, and difference-based agreement assessment.

To assess the agreement between the vision-based method and the GymAware, the following statistical analyses were applied to the paired data synchronously collected by both methods. All statistical analyses were performed using Python 3.12.7 (utilizing the pingouin, scipy, and matplotlib libraries), with the significance level (α) set at 0.05.

#### 2.5.1. Data Preprocessing

Data pairs from the vision-based method and the GymAware measurement of the same landmine press movement were extracted for subsequent analysis. No data points were excluded as outliers unless a clear recording error (e.g., video loss or sensor detachment) was identified. Data are presented as Mean ± SD.

#### 2.5.2. Statistical Analysis Methods

The Pearson correlation coefficient (r) quantified the linear correlation between the vision-based method and GymAware. Correlation strength was interpreted as [19]: r ≥ 0.9 (very strong), 0.7 ≤ r < 0.9 (strong), 0.5 ≤ r < 0.7 (moderate), 0.3 ≤ r < 0.5 (weak), and r < 0.3 (very weak).

Since both measurement methods contain inherent error, Deming regression was employed instead of ordinary least squares regression to evaluate systematic differences between them. This method accounts for measurement error in both variables and provides unbiased estimates of the regression slope and intercept [20]. Assuming both methods had similar error variance (λ = 1), we determined fixed bias by testing if the intercept significantly differed from zero, and proportional bias by testing if the slope significantly differed from one. We reported 95% confidence intervals for both to facilitate statistical inference.

Bland–Altman analysis assessed agreement between methods by plotting differences (vision-based minus GymAware) against their mean [21]. The mean difference with its 95% CI estimated average bias. The limits of agreement (LoAs), defined as the Mean ± 1.96 × SD, described the range within which 95% of the differences between the two methods were expected to lie. The 95% CIs for the LoAs were also calculated. A paired *t*-test was used to evaluate whether a statistically significant mean difference existed between the two methods.

Cohen’s d effect size was computed to quantify the magnitude of the mean difference between methods, independent of sample size. Effect sizes were interpreted as follows [20]: |d| < 0.2 (negligible), 0.2 ≤ |d|< 0.5 (small), 0.5 ≤ |d| < 0.8 (medium), and |d| ≥ 0.9 (large).

In interpreting the results, statistical significance (*p* < 0.05) was considered alongside effect sizes and the width of the LoAs to determine whether observed differences were practically meaningful in the context of strength training monitoring [21,22].

## 3. Results

### 3.1. Results of the Object Detection Model Based on Enhanced YOLOv8-OBB

All model performance metrics reported in this section are based on the test set described in Section 2.1, under controlled gym conditions with fixed camera placement and consistent lighting.

#### 3.1.1. Model Training Convergence Results

The Enhanced YOLO-OBB was trained for 120 epochs. The training convergence curves, including bounding box loss, classification loss, and Distribution Focal Loss, are provided in Figure S1 (Supplementary Materials). On the training set, the bounding box loss decreased consistently from 3.10 to 0.93, the classification loss declined from 4.09 to 0.45, and the Distribution Focal Loss reduced from 4.09 to 1.37. Validation loss trends aligned with training, with final values converging to 0.50 (bounding box), 0.38 (classification), and 1.65 (Distribution Focal Loss).

#### 3.1.2. Model Detection Performance

**Figure 3:**
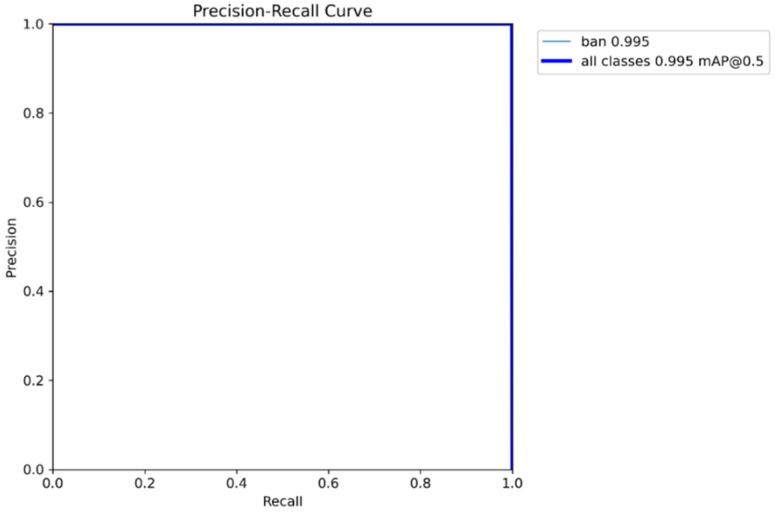
Precision–recall curve. Note that the precision-recall curves for the ‘ban’ class and ‘all classes’ are identical, resulting in a single visible line.

The precision–recall curve of the proposed model on the test set is shown in Figure 3.

The model achieved a mean average precision (mAP@0.5) of 0.995, a maximum precision of 1.00, a maximum recall of 1.00, and an area under the precision–recall curve (AUC) of 0.992.

For detailed classification performance, the normalized confusion matrix is available in Figure S2 (Supplementary Materials). The model achieved a recall of 1.00 and a precision of 1.00 for the “fixed” category, with a background false detection rate of <0.05 and an overall classification accuracy of >0.98.

#### 3.1.3. Model Robustness Across Confidence Threshold

The confidence-dependent performance curves (F1–confidence and precision–confidence) are presented in Figure S3 (Supplementary Materials). The maximum F1-score was 1.00. Across a broad confidence threshold range (0.0 to 0.8), the F1-score consistently remained at or above 0.95, with a low standard deviation of 0.02, indicating stable performance.

The average precision across the entire confidence range was 0.998. Precision maintained a value of 1.00 in the high-confidence interval (>0.7) and never dropped below 0.985 in the low-to-medium-confidence intervals.

These results demonstrate that the enhanced YOLOv8-OBB model achieved high detection accuracy and robustness under the experimental conditions of this study.

### 3.2. Results of the Method Comparison Experiment

The following agreement results are derived from the method comparison experiment described in Section 2.4, conducted under controlled conditions with fixed camera placement, consistent lighting, and standardized loading protocols. The findings should be interpreted within these experimental constraints.

#### 3.2.1. Kinematic Indicators

Four kinematic indicators (peak velocity, mean velocity, peak power, and mean power) were collected from a total of 247 valid landmine press trials using both methods. These trials were performed by 24 participants across four incremental loads (20 kg, *n* = 72; 25 kg, *n* = 63; 30 kg, *n* = 56; 35 kg, *n* = 56). Table 1 summarizes the descriptive statistics (mean ± SD) for each indicator across all loads. As shown, both systems captured similar trends across loads, with velocity decreasing and power generally increasing as the load increased. However, the vision-based system consistently yielded higher velocity estimates compared to GymAware across all conditions.

**Figure 4:**
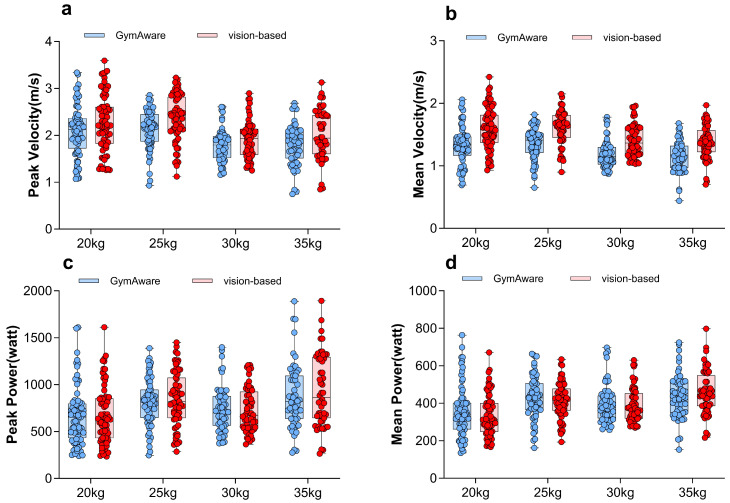
Four kinematic indicators across the two methods and across four loads. Blue dots represent measurements from the GymAware system, and red dots represent measurements from the vision-based system. (**a**) peak velocity, (**b**) mean velocity, (**c**) peak power, (**d**) mean power. Error bars represent standard deviation.

Figure 4 visually compares the four kinematic indicators between the two systems grouped by load. The plots confirm the systematic tendency of the vision-based method to estimate higher velocities, while power outputs show closer alignment, particularly at heavier loads.

#### 3.2.2. Bland–Altman Analysis

Bland–Altman analysis was performed to assess the agreement between the two methods by quantifying the mean bias and the 95% limits of agreement (LoAs). The results, summarized in Table 2, indicate that the vision-based method systematically overestimated peak and mean velocity across all loads, with mean biases of +0.19 m/s and +0.23 m/s, respectively. The width of the LoAs for velocity metrics was relatively narrow, suggesting consistent bias. For power metrics, the mean biases were smaller in magnitude and often not statistically significant, with the LoAs narrowing notably at higher loads (mean power LoA reduced from 248 W at 20 kg to 153 W at 35 kg).

**Figure 5:**
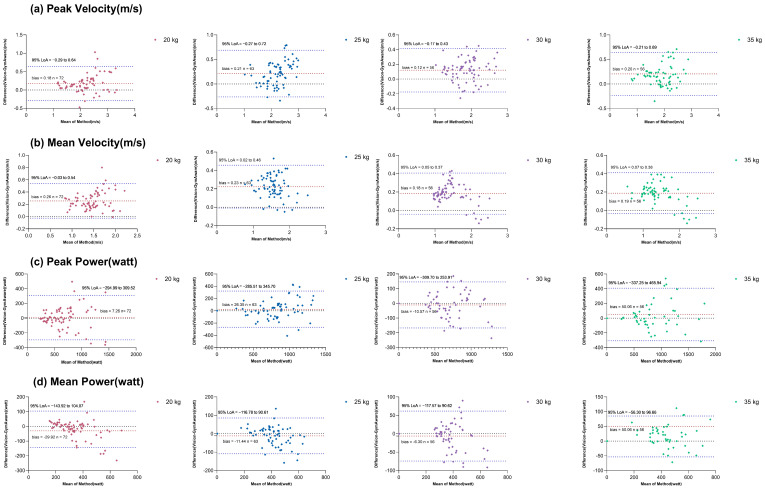
Bland–Altman plots for (**a**) peak velocity, (**b**) mean velocity, (**c**) peak power, (**d**) mean power across all loads. Red dashed line: mean bias; blue dashed lines: 95% limits of agreement; black dashed line: the line of perfect agreement (zero difference between methods).

The corresponding Bland–Altman plots are presented in Figure 5. The plots illustrate that the differences between methods are generally evenly distributed around the mean bias line without obvious patterns of heteroscedasticity, except for a slight increase in scatter for peak power at the highest load.

#### 3.2.3. Agreement and Correlation Analysis

A comprehensive agreement assessment was conducted using Pearson correlation, paired-sample *t*-tests, Cohen’s d effect sizes, and Deming regression. The results are summarized in Table 3. While very strong Pearson correlations (r ≥ 0.85) were observed for all indicators, the presence of statistically significant mean biases (*p* < 0.05 for most velocity comparisons) and large effect sizes (e.g., Cohen’s d > 0.8 for velocity) indicates that the two methods should not be considered interchangeable without correction. The Deming regression slopes for velocity were consistently greater than one (range: 1.05–1.27), confirming a proportional overestimation by the vision-based system.

In summary, the two systems exhibited strong linear correlations across all measured kinematic indicators. However, the vision-based method systematically overestimated linear velocity by approximately 9.8% (peak) to 18.4% (mean), a bias attributable primarily to the difference in effective measurement radius as explained in Section 2.3.1. Power metrics showed better agreement, with mean biases often non-significant and LoAs narrowing under heavier loads, suggesting improved comparability in high-load conditions.

## 4. Discussion

This study developed and evaluated a vision-based system for monitoring landmine press kinematics using an enhanced YOLOv8-OBB detection model and a simplified biomechanical model. The discussion is structured into three parts: model performance and robustness, agreement between measurement systems and the practical implications, and limitations with future directions.

### 4.1. Model Performance and Robustness

**Figure 6:**
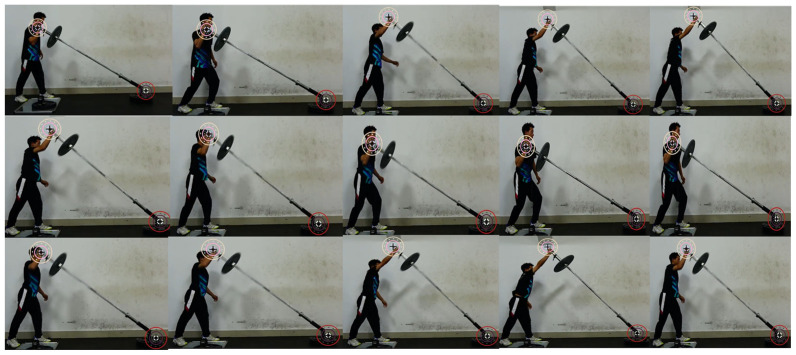
Model detection performance on a new task sequence, demonstrating robust generalization under movement.

The proposed Enhanced YOLOv8-OBB model demonstrated excellent detection accuracy (mAP@0.5 = 0.995) and robust performance across confidence thresholds on the test set under controlled conditions (Figure 3 and Figure S3). This high performance can be attributed to three key architectural improvements tailored for small-object detection in dynamic environments: the polarized self-attention (PSA) module for filtering distracting backgrounds, the C3k2 module for capturing a wide range of angular variations through heterogeneous feature fusion, and the SPPF module for efficient motion context understanding. As shown in Figure 6, the model successfully generalizes to new task sequences, accurately tracking both barbell ends despite athlete motion and varying loads. This capability is crucial for a markerless system intended for training environments, where robustness to visual clutter and motion blur is essential. The results confirm that with targeted enhancements, object detection models like YOLOv8 can be adapted to create reliable “vision sensors” for specialized biomechanical-tracking tasks.

### 4.2. Agreement and Practical Implication

**Figure 7:**
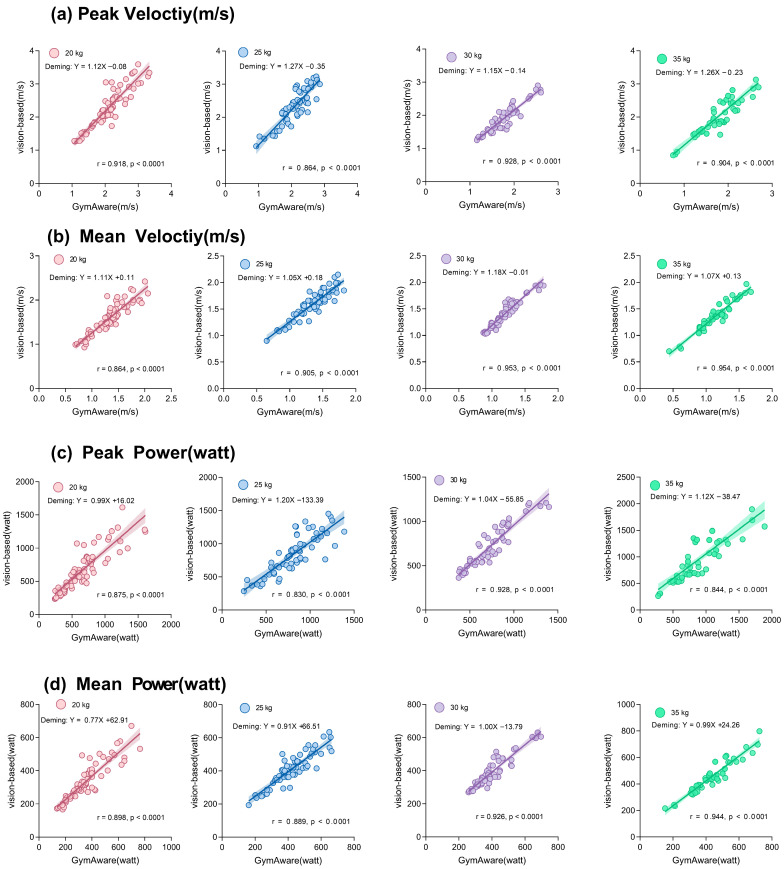
Correlation and Deming regression plots for (**a**) peak velocity, (**b**) mean velocity, (**c**) peak power and (**d**) mean power. Solid line: Deming regression fit.

The method comparison study revealed a strong linear relationship between the vision-based system and the GymAware linear transducer across all loads and kinematic metrics, yet also identified predictable systematic differences. The vision-based method consistently produced higher linear velocity estimates (Figure 7). This systematic bias is primarily attributed to the difference in the effective measurement radius between the two systems (2.20 m vs. 2.05 m), as established in Section 2.3.1. Furthermore, the vision system’s use of lighter filtering to preserve peak information, while GymAware’s stronger low-pass filtering to mitigate integration drift may also contribute to the observed overestimation.

For power metrics, the agreement between systems improved as load increased, as shown by the narrowing limits of agreement (Table 2, Figure 5). This pattern indicates that under high loads, GymAware is prone to electromechanical noise and drift [5,7], while the vision-based method provides more stable measurements. This stability arises because the vision system calculates kinematics from image coordinates of both barbell ends, avoiding the cumulative errors common in cable displacement sensors during forceful, complex movements. These findings indicate that the vision-based system provides a practical and markerless alternative for monitoring landmine press kinematics.

### 4.3. Limitations and Future Directions

This study provides positive evidence for the vision-based approach, but has several limitations that should guide future research. First, the agreement validation relied on GymAware as a comparator. While GymAware is widely used, it is not a gold standard for diagonal, rotational movements like the landmine press [11]. Future work should employ a higher-validation reference system, such as 3D motion capture systems [23] and force plates [24], to establish the absolute accuracy of the vision-based method.

Second, the biomechanical model employed several simplifying assumptions (Section 2.3.1), treating the barbell as a rigid body in planar rotation and neglecting grip micro-motions and out-of-plane components. Consequently, the reported force and power outputs are model-based estimates. While these assumptions are common for practical monitoring and strong agreement with a commercial device, they define the boundaries of the model’s applicability.

Third, model performance and agreement were evaluated under controlled conditions with fixed camera angles and consistent lighting. The system’s robustness to variable field conditions, such as different camera viewpoints, occlusions, or changing lighting, remains to be evaluated. Future studies should validate the framework across a wider range of practical training environments to assess its true generalizability.

Despite these limitations, the proposed system demonstrates several practical advantages over traditional LPT sensors. It is markerless, eliminating the need for attaching devices to the athletes or equipment to reduce setup time and avoid interference with natural movement patterns. The system relies on affordable and widely available consumer-grade cameras, making it accessible for use in a variety of settings. Furthermore, the vision-based pipeline is fundamentally immune to the electromechanical drift and cable attachment that can affect linear transducers, especially under high loads or dynamic conditions.

## 5. Conclusions

This study developed and evaluated a CV-based kinematic measurement system for the landmine press using an enhanced YOLOv8-OBB object detection model and simplified biomechanical mathematical model. The system enables markerless, non-contact measurement of velocity and power indicators during the concentric phase of the movement. Under the controlled experimental conditions described, the main findings can be summarized as follows:

1. The enhanced detection model achieved high accuracy and robustness in tracking the moving and fixed ends of the barbell, with a mean average precision (mAP@0.5) of 0.995 on the test set. The introduced architectural improvements, including a polarized self-attention mechanism, an improved C3k2 module, and an optimized SPPF structure, enabled reliable performance in a controlled, dynamic gym environment.
2. The vision-based method showed strong agreement with the GymAware linear transducer across four loads. The observed systematic differences in linear velocity primarily stem from a fundamental methodological divergence: the vision system employs a simplified yet practical planar-rotation model based on the full barbell length, whereas the GymAware estimates displacement from a fixed cable attachment point. Despite this difference in underlying mechanics, the two methods exhibited strong correlations (r > 0.85) for all kinematic indicators, indicating that the vision-based model captures the essential dynamics of the movement. Furthermore, agreement in power metrics improved with increasing load, suggesting that the vision-based approach can provide stable and practical measurements, particularly under high-load conditions where cable-based sensors are more susceptible to electromechanical drift and noise.
3. The proposed framework offers a practical, markerless alternative for monitoring barbell exercises. It eliminates the need for attached sensors, relies on affordable camera hardware, and can be deployed in both laboratory and field settings without interfering with the athlete’s movement. The systematic bias identified is consistent and can be corrected if direct comparability with sensor-based devices is required.

This study demonstrates that computer vision combined with a simplified biomechanical model can deliver reliable, markerless monitoring of a landmine press. The methodology is adaptable to other barbell exercises through model retraining, and future integration of multi-view cameras could enhance its accuracy through 3D motion reconstruction. By providing an accessible and affordable tool for movement analysis, this study contributes to intelligent, vision-based training environments.

## Figures and Tables

**Table 1:** Kinematic indicators (mean ± SD) between the two methods across four loads.

Load	*n*	Method	Peak Velocity (m/s)	Mean Velocity (m/s)	Peak Power (m/s)	Mean Power (m/s)
all	247	vison-based	2.16 ± 0.54	1.50 ± 0.30	789.41 ± 324.67	395.01 ± 115.08
		GymAware	1.96 ± 0.47	1.27 ± 0.27	771.35 ± 299.11	402.64 ± 124.50
20 kg	72	vison-based	2.27 ± 0.60	1.59 ± 0.34	678.80 ± 307.05	335.91 ± 110.01
		GymAware	2.09 ± 0.54	1.33 ± 0.31	671.54 ± 310.59	355.83 ± 139.64
25 kg	63	vison-based	2.36 ± 0.50	1.61 ± 0.26	849.45 ± 288.61	414.39 ± 95.57
		GymAware	2.13 ± 0.41	1.36 ± 0.25	819.36 ± 248.07	427.48 ± 114.82
30 kg	56	vison-based	1.92 ± 0.40	1.40 ± 0.25	715.20 ± 251.24	386.54 ± 103.44
		GymAware	1.789 ± 0.36	1.19 ± 0.22	743.09 ± 243.63	400.02 ± 103.37
35 kg	56	vison-based	2.03 ± 0.53	1.38 ± 0.27	938.30 ± 380.04	457.65 ± 116.38
		GymAware	1.79 ± 0.43	1.16 ± 0.25	873.95 ± 345.93	437.47 ± 117.41

**Table 2:** Bland–Altman analysis between the two methods across four loads.

Load	Kinematic Indicators	Mean Variation	95% LoA
all	Peak Velocity	0.19	−0.25 to 0.64
	Mean Velocity	0.23	0.02 to 0.45
	Peak Power	18.06	−312.52 to 348.63
	Mean Power	−7.63	−116.18 to 100.92
20 kg	Peak Velocity	0.18	−0.29 to 0.64
	Mean Velocity	0.26	−0.03 to 0.54
	Peak Power	7.26	−294.99 to 309.52
	Mean Power	−19.92	−143.92 to 104.07
25 kg	Peak Velocity	0.23	−0.27 to 0.72
	Mean Velocity	0.24	0.02 to 0.46
	Peak Power	30.10	−285.51 to 345.70
	Mean Power	−13.09	−116.78 to 90.61
30 kg	Peak Velocity	0.13	−0.17 to 0.43
	Mean Velocity	0.21	0.05 to 0.37
	Peak Power	−27.90	−309.70 to 253.91
	Mean Power	−13.48	−117.57 to 90.62
35 kg	Peak Velocity	0.24	−0.21 to 0.69
	Mean Velocity	0.22	0.07 to 0.38
	Peak Power	64.35	−337.25 to 465.94
	Mean Power	20.18	−56.30 to 96.66

**Table 3:** Assessment of agreement between the two methods across different loads.

Load	Kinematic Indicators	r	*p*-Value	Cohen’s d	Deming Slope	Deming Intercept
all	Peak Velocity	0.91	0.00	0.86	1.18	−0.15
	Mean Velocity	0.86	0.00	2.10	1.14	0.05
	Peak Power	0.86	0.09	0.11	1.10	−59.38
	Mean Power	0.90	0.03	−0.14	0.92	26.20
20 kg	Peak Velocity	0.92	0.00	0.74	1.12	−0.08
	Mean Velocity	0.91	0.00	1.78	1.11	0.11
	Peak Power	0.88	0.69	0.05	0.99	16.02
	Mean Power	0.90	0.01	−0.32	0.77	62.91
25 kg	Peak Velocity	0.87	0.00	0.90	1.27	−0.35
	Mean Velocity	0.91	0.00	2.16	1.05	0.18
	Peak Power	0.83	0.14	0.19	1.20	−133.39
	Mean Power	0.89	0.05	−0.25	0.81	66.51
30 kg	Peak Velocity	0.93	0.00	0.87	1.15	−0.14
	Mean Velocity	0.94	0.00	2.54	1.18	−0.01
	Peak Power	0.83	0.15	−0.19	1.04	−55.85
	Mean Power	0.87	0.06	−0.25	1.00	−13.79
35 kg	Peak Velocity	0.90	0.00	1.04	1.26	−0.23
	Mean Velocity	0.95	0.00	2.78	1.07	0.13
	Peak Power	0.85	0.02	0.31	1.12	−38.47
	Mean Power	0.94	0.00	0.52	0.99	24.26

## Data Availability

The datasets generated and/or analyzed during the current study are available in the Zenodo [https://doi.org/10.5281/zenodo.18598087].

## Supplementary Materials

The following supporting information can be downloaded at: https://www.mdpi.com/article/10.3390/s26041161/s1, Figure S1: Training Loss and Evaluation Metrics Curves; Figure S2: Confusion Matrix Normalized; Figure S3: (a) F1-Confidence Curve, (b) Precision-Confidence Curve.
