# Supplementary material for: Landmine Press Kinematics Measured with an Enhanced YOLOv8 Model and Mathematical Modeling
Source: Sensors (Basel). 2026 Feb 11;26(4):1161. doi: 10.3390/s26041161 (PMC12944738; doi:10.3390/s26041161)
Supplement: Supplementary file 1 [file sensors-26-01161-s001.zip › sensors-4092319-supplementary.pdf]

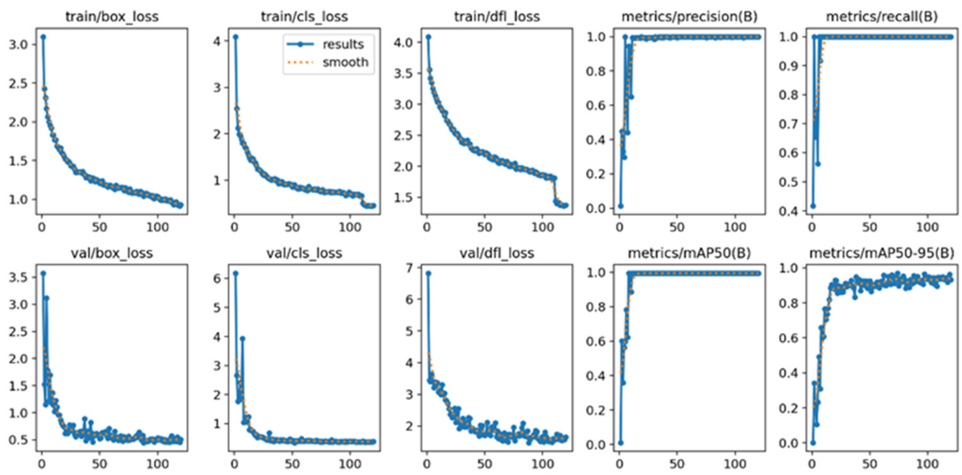

Figure S1. Training Loss and Evaluation Metrics Curves

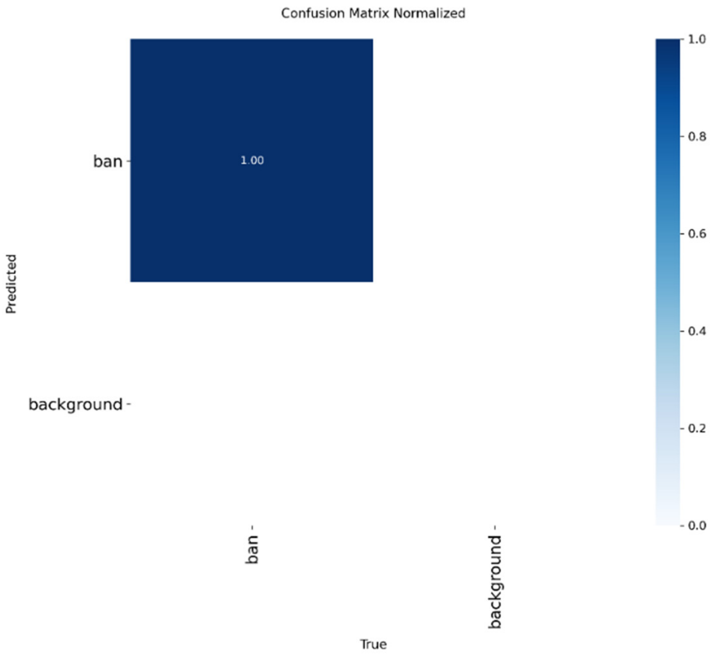

Figure S2. Confusion Matrix Normalized

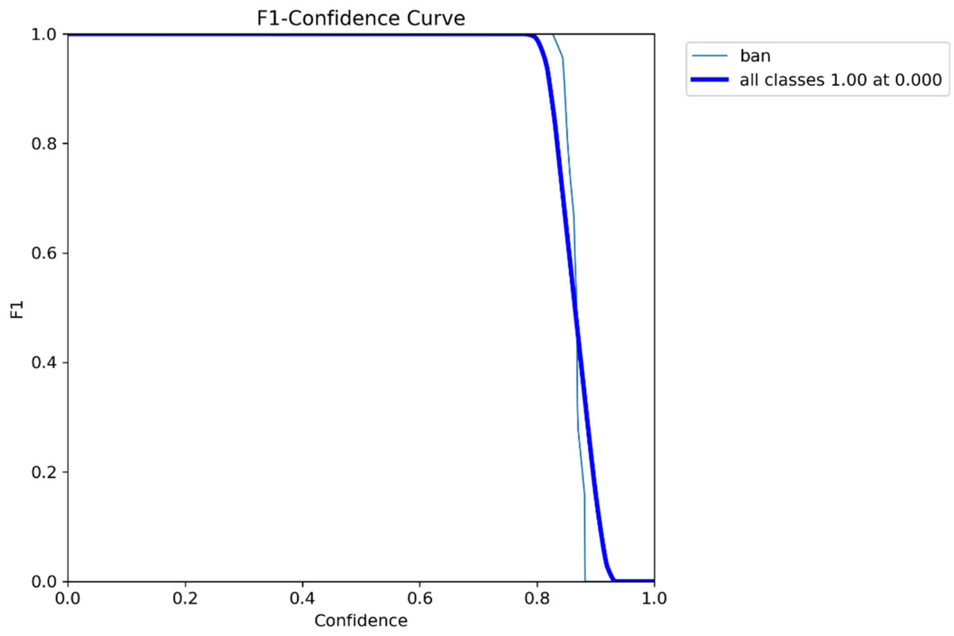

Figure S3a. F1-Confidence Curve

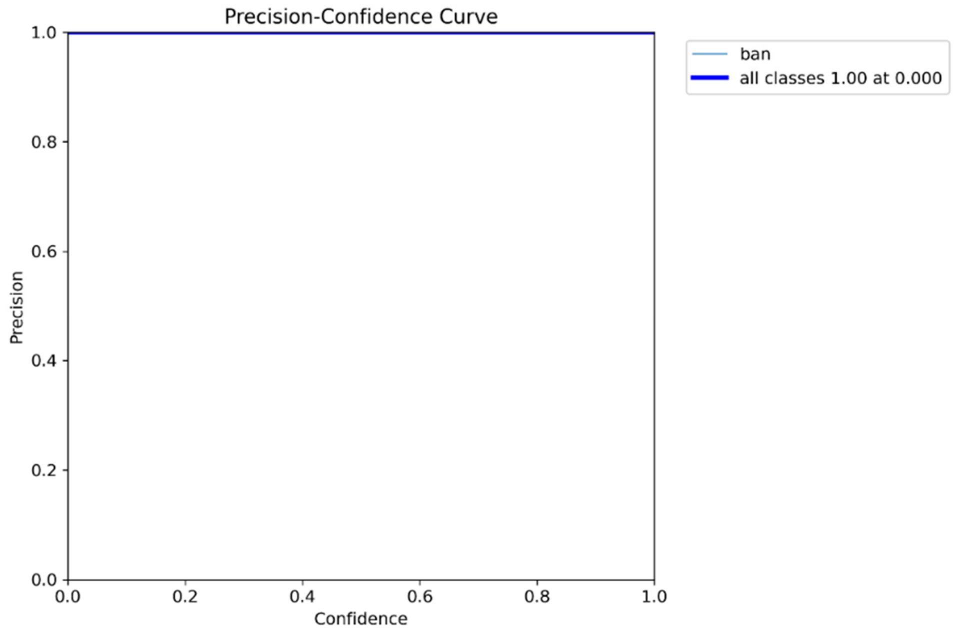

Figure S3b. Precision-Confidence Curve
